# *Notes from the Field*: Pharmacy Needs After a Natural Disaster — Puerto Rico, September–October 2017

**DOI:** 10.15585/mmwr.mm6713a4

**Published:** 2018-04-06

**Authors:** Amy M. Lavery, Anita Patel, Tegan K. Boehmer, Leslie Lee, Tina Bhavsar, Jacqueline Thomas, Lori Hall, Suzanne Beavers, Maria Murray, Satish K. Pillai

**Affiliations:** ^1^Epidemic Intelligence Service, CDC; ^2^Geospatial Research Analysis and Services Program, Office of Environmental Health Emergency Management, National Center for Environmental Health/Agency for Toxic Substances and Disease Registry, CDC; ^3^Office of the Director, National Center for Immunization and Respiratory Diseases, CDC; ^4^Division of Emergency and Environmental Health Services, National Center for Environmental Health, CDC; ^5^Division of Strategic National Stockpile, Office of Public Health Preparedness and Response, CDC; ^6^Division of Environmental Hazards and Health Effects, National Center for Environmental Health, CDC; ^7^IQVIA Government Solutions, Durham, North Carolina; ^8^Division of Preparedness and Emerging Infections, National Center for Emerging and Zoonotic Infectious Diseases, CDC.

After disasters such as hurricanes, access to prescription drugs might be limited or inaccessible. For example, after Hurricane Ivan made landfall near Mobile, Alabama, in 2004, an assessment of its impact on pharmacies in the affected areas found that 53% had depleted supplies and at least 26% had to prioritize distribution to patients because of limited supplies (*1*). A 2005 study of Hurricane Katrina evacuees in San Antonio, Texas, found that disaster medical assistance teams were more prepared to provide for acute than chronic illnesses although more than two thirds (68%) of patients requested drugs to treat chronic conditions (*2*). Understanding the prescribing practices of a region can inform post-disaster medication needs and planning for future emergencies.

On September 20, 2017, Hurricane Maria made landfall in Puerto Rico as a Category 4 hurricane. Five days later, only approximately 29% of pharmacies reporting to Healthcare Ready, an organization that provides information on access to pharmacies during an emergency, were open (*3*). CDC summarized data within the IQVIA data source (formerly IMSHealth, QuintilesIMS)* to supply the U.S. Department of Health and Human Services emergency response team with projections of formulary health care needs following Hurricane Maria. Prescription data can also highlight important chronic disease concerns for a community.

The IQVIA database contains information on drugs dispensed by retail facilities and is normally used by industry to monitor drug use and trends in the market. Information on the top 200 drugs dispensed by retail facilities in Puerto Rico during June–August was abstracted from the database. An average of total prescriptions for these 200 drugs for the 3-month period was calculated. The top 200 drugs accounted for approximately 80% of all prescription drugs dispensed in retail facilities. Drugs were categorized by therapeutic category and administration route (e.g., oral, inhalation, or topical) by a team of clinicians at CDC.

During June–August 2017, the top categories of drugs prescribed were for cardiovascular (average = 21% of prescriptions filled), psychiatric (12%), and analgesic (10%) drugs (Table). Among the cardiovascular drugs prescribed, a majority were angiotensin II receptor antagonists (29%), beta blockers (20%), and angiotensin-converting enzyme (ACE) inhibitors (18%). The most frequently dispensed individual drugs were thyroid replacement hormones (230,324 prescriptions dispensed, 5% of total dispensed), gabapentin (144,114 prescriptions dispensed, 3% of total), and metformin (141,734 prescriptions dispensed, 3% of total). Ninety percent of prescribed drugs were for oral administration.

**TABLE:** Top therapeutic categories for retail-dispensed prescriptions — Puerto Rico, June–August, 2017*

Therapeutic category	No. (%) prescriptions
Cardiovascular	971,234 (20.7)
Psychiatric	554,839 (11.8)
Analgesic^†^	449,532 (9.6)
Lipid lowering	349,533 (7.5)
Diabetes	346,104 (7.4)
Gastrointestinal	342,146 (7.3)
Neurologic	287,038 (6.1)
Thyroid	234,982 (5.0)
Antibiotics	225,009 (4.8)
Pulmonary	150,525 (3.2)
Other^§^	781,241 (16.6)
**Total**	**4,692,183 (100.0)**

* Categorizations represent the therapeutic categories for the top 200 dispensed medications, information about which was abstracted from the IQVIA database for this analysis. The top 200 dispensed medications account for approximately 80% of the total prescriptions dispensed in Puerto Rico.

^†^ Includes prescriptions for narcotics and other medications used for pain management (e.g., pregabalin, acetaminophen, phenyltoloxamine, and tramadol).

^§^ Includes antihistamines, diuretics, muscle relaxants, nutritional supplements, ophthalmic solutions, medications for enlarged prostate or benign prostatic hyperplasia, rheumatologic, steroids, and topical creams.

The distribution of pharmaceutical dispensing practices identified using the IQVIA database can provide information for planning both before and after a disaster. The most frequently prescribed drugs help focus immediate supply measures for response and recovery efforts, supporting a vital public health need. The IQVIA database used in this analysis is limited to retail facilities and does not include hospitals or other institutions such as nursing homes. Furthermore, some critical drugs might not be represented in this data set, including insulin, which can also be purchased over the counter; hence, some of the prescribed quantities in this data set could be an underestimate of medication needs. Although insulin was not a most frequently purchased or prescribed drug, it is a daily need for persons with insulin-dependent diabetes and should be prioritized. To have a more complete picture of important drugs that might be needed after a disaster, multiple data sources, including drug sales data to hospitals, clinics, and nursing homes, as well as information provided by third-party claims adjudication data, could be analyzed to inform public health activities and guide collaborations with drug suppliers to respond to and recover from large-scale disasters.
